# Changes of Prostate-Specific Membrane Antigen-Radioligand Uptake on PET with Systemic Therapy in Patients with Metastatic Renal Cell Carcinoma

**DOI:** 10.3390/cancers17111736

**Published:** 2025-05-22

**Authors:** Sophie Carina Kunte, Adrien Holzgreve, Marcus Unterrainer, Josef Zahner, Hans Peter Schmid, Magdalena Schöll, Iulia Blajan, Gabriel T. Sheikh, Dirk Mehrens, Jozefina Casuscelli, Alexander J. Tamalunas, Rudolf A. Werner, Christian G. Stief, Michael Staehler, Lena M. Unterrainer

**Affiliations:** 1 Department of Nuclear Medicine, LMU University Hospital, LMU Munich, 81377 Munich, Germanymarcus.unterrainer@die-radiologie.de (M.U.); josef.zahner@med.uni-muenchen.de (J.Z.); hans.schmid@med.uni-muenchen.de (H.P.S.); magdalena.schoell@med.uni-muenchen.de (M.S.); gabriel.sheikh@med.uni-muenchen.de (G.T.S.); rudolf.werner@med.uni-muenchen.de (R.A.W.); lena.unterrainer@med.uni-muenchen.de (L.M.U.); 2 Bavarian Cancer Research Center (BZKF), Partner Site Munich, 80539 Munich, Germany; 3 Ahmanson Translational Theranostics Division, David Geffen School of Medicine, University of California Los Angeles (UCLA), Los Angeles, CA 90095, USA; 4 Die RADIOLOGIE, 80331 Munich, Germany; 5 Department of Urology, LMU University Hospital, LMU Munich, 81377 Munich, Germany; iulia.blajan@med.uni-muenchen.de (I.B.); jozefina.casuscelli@med.uni-muenchen.de (J.C.); alexander.tamalunas@med.uni-muenchen.de (A.J.T.); christian.stief@med.uni-muenchen.de (C.G.S.); michael.staehler@med.uni-muenchen.de (M.S.); 6 Department of Radiology, LMU University Hospital, LMU Munich, 81377 Munich, Germany; 7 The Russell H Morgan Department of Radiology and Radiological Sciences, Division of Nuclear Medicine, Johns Hopkins School of Medicine, Baltimore, MD 21205, USA

**Keywords:** metastatic renal cell carcinoma (mRCC), kidney cancer, [^18^F]PSMA-1007 PET/CT, systemic therapy (TKI/CI), SUV

## Abstract

**Background/Objectives**: Early treatment assessment in metastatic renal cell carcinoma (mRCC) remains challenging due to the limited accuracy of current imaging methods. Given prostate-specific membrane antigen (PSMA) overexpression in mRCC, PSMA PET is a promising approach. Despite numerous studies on PSMA imaging in mRCC, data on PSMA uptake changes during systemic therapy are scarce. We analyzed PSMA uptake on PET after treatment initiation in mRCC patients. **Methods**: A retrospective single-center analysis of mRCC patients who underwent [^18^F]PSMA-1007 PET/CT before (PET_1_) and at a mean of 9.5 weeks after (PET_2_) starting systemic therapy was conducted. PSMA uptake in metastatic lesions was compared by region and RCC subtype. Uptake differences between PET_1_ and PET_2_ were analyzed using an unpaired *t*-test. **Results**: This study included 25 patients (mean age 65.2 ± 14.7 years; 20 male) with mRCC. A total of 113 (PET_1_) and 48 (PET_2_) metastases were assessed. Lymph node metastases showed stable PSMA uptake (median SUV_max_) after treatment (7.8 vs. 7.7, *p* = 0.77), while uptake by bone (6.4 vs. 12.4, *p* = 0.03) and lung metastases (4.5 vs. 8.1, *p* = 0.004) increased significantly. SUV stability in lymph nodes was independent of RCC subtype (ccRCC: *p* = 0.48, pRCC: *p* > 0.99). Bone (6.6 vs. 15.9, *p* = 0.008) and lung metastases (4.8 vs. 8.1, *p* = 0.02) had higher PSMA uptake in ccRCC, unlike pRCC (bone: 6.2 vs. 6.0, *p* = 0.86). **Conclusions**: Alterations of PSMA-radioligand uptake are seen in bone and pulmonary metastases but not in lymph node metastases after initiation of systemic treatment in patients with mRCC. ccRCC has a higher PSMA uptake than other RCC subtypes.

## Simple Summary

Due to PSMA overexpression, PSMA PET showed promising results in the imaging of mRCC. However, data on PSMA uptake changes during systemic therapy are scarce. This study analyzed PSMA uptake changes on PSMA PET after initiation of systemic therapy in mRCC patients. Our results demonstrated distinct uptake changes in mRCC metastases on PSMA PET, with increases in bone and lung lesions but stable lymph node uptake after initiation of systemic therapy. ccRCC further showed a higher PSMA uptake than other RCC subtypes. This highlights the potential of PSMA PET for therapy monitoring aswell as subtype-specific PSMA uptake characteristics in mRCC.

## 1. Introduction

Renal carcinoma (RCC) is the most prevalent type of renal tumor and encompasses a heterogenous group of histological subtypes [1]. The majority of RCC diagnoses result from incidental findings on magnetic resonance imaging (MRI), computed tomography (CT) or ultrasonography. While conventional imaging provides anatomical information, nuclear medicine examinations enables the detection of molecular tumor characteristics [2]. The most utilized radioligand in the context of oncological malignancies is 2-[^18^F]fluoro-2-deoxy-D-glucose ([^18^F]FDG), which is employed in positron emission tomography (PET)/CT imaging. However, [^18^F]FDG-PET/CT has a limited utility in RCC due to the variable and often low FDG-avidity of RCC lesions [3,4]. Therefore, different radiotracers are required to improve lesion detection and monitoring of patients undergoing systemic therapy [5,6,7].

Carboxypeptidase type II (prostate-specific membrane antigen—PSMA) is a transmembrane protein that is not only expressed on the surface of prostate cancer cells but also in neovascular endothelial cells of various solid malignancies, including RCC [8,9]. Since RCC is typically hypervascular, PSMA PET imaging emerges as a promising imaging modality, irrespective of glucose metabolism [10,11,12,13,14]. Recent studies have indicated that PSMA PET imaging may have a superior capacity to detect RCC lesions when compared with CT or [^18^F]FDG PET [15]. A preliminary analysis of our group indicated that PSMA PET provides complementary or even superior information on disease extent in patients with metastatic RCC (mRCC) compared to CT alone [16,17,18]. Notwithstanding these encouraging data, the impact of systemic therapies, such as tyrosine kinase inhibitors (TKI) or checkpoint inhibitors (CI), on tracer uptake into metastases remains to be elucidated. Understanding these mechanisms is essential in order to determine the value of PSMA PET as a biomarker for therapy monitoring and response assessment.

However, data on changes of PSMA-radioligand uptake in metastases of patients with mRCC under systemic therapy are lacking. To date, it remains unclear whether and to what extent PSMA uptake changes occur in different organ systems in mRCC patients, what biological mechanisms underlie these changes, and how such findings should be interpreted in the context of disease monitoring or treatment response.

To address this gap and explore its potential implications for treatment response assessment, we investigated PSMA-radioligand uptake dynamics across different histological subtypes of mRCC under systemic therapy.

## 2. Materials and Methods

### 2.1. Study Design and Study Population

This retrospective single-center analysis was approved by the institutional ethics committee of the Ludwig Maximilians University Munich (IRB#22-1076). Patients were consecutively included if they met the following criteria: (1) a histologically proven RCC with evidence of metastases (M1) on conventional imaging, (2) systemic therapy with tyrosine kinase (TKI) or checkpoint inhibitor therapy (CI), and (3) an [^18^F]PSMA-1007 PET/CT prior to and after the initiation of therapy.

PSMA-radioligand uptake was evaluated separately on PET prior to therapy initiation (PET_1_) and at a mean of 9.5 weeks after initiating systemic therapy (PET_2_).

### 2.2. Radiopharmaceutical and Imaging Protocol

All radiotracer syntheses were performed in accordance with Good Manufacturing Practice standards. In order to minimize inter-batch variability, standardized production protocols were employed, and consistent quality control parameters were ensured across all synthesis batches utilized in this study.

Patients received a median activity of 163 MBq (range 114–263 MBq, adjusted for body weight) of [^18^F]PSMA-1007 intravenously per PET scan, in accordance with previously reported radiosynthesis and administration procedures [19]. Furthermore, furosemide (20 mg) was administered to all patients without contraindications [20]. The radiopharmaceutical was administered to each patient individually in accordance with the provisions of the German Medicinal Products Act, §13(2b). The PET/CT was conducted in accordance with the previously described methodology [16]. In brief, the PET acquisition was started 60 min after the injection of the tracer and lasted 20 min. Furthermore, a contrast-enhanced CT scan in the portal–venous phase from the skull base to the mid-thigh was conducted.

### 2.3. [^18^F]PSMA-1007 PET Analysis

Image analysis was conducted by S.C.K. and L.M.U. Any focal tracer uptake that was higher than the surrounding background and not associated with known sites of non-tumorous signal (e.g., urinary excretion, physiological uptake in normal organs) was considered suspicious for malignancy. The PSMA-radioligand uptake of all single metastases per organ system (lymph node, bone, lung, liver, other including thyroid gland, adrenal gland, peritoneal lesions and pancreas) was analyzed in PET_1_ and PET_2_.

Due to the retrospective nature of the study and limitations in lesion detectability and size, no direct lesion-to-lesion pairing between PET_1_ and PET_2_ was performed. Conversely, mean uptake values (SUV_max_, SUV_mean_) were ascertained for predefined organ systems per patient. Subsequent comparisons between PET_1_ and PET_2_ were conducted at the organ level.

### 2.4. Statistical Analysis

Statistical analysis was performed with GraphPad Prism (Version 9.4.0.673, GraphPad Software, Boston, MA, USA). Descriptive statistics are displayed as mean  ±  standard deviation or median and interquartile range (1st quartile: Q1; 3rd quartile: Q3). A Shapiro–Wilk normality test was conducted to test for normality. Differences in the aggregated uptake per organ system between PET_1_ and PET_2_ were analyzed using an unpaired *t*-test. A two-tailed *p*-value < 0.05 was considered statistically significant.

## 3. Results

### 3.1. Study Population

A total of 25 mRCC patients were included in this analysis (mean age 65.2 ± 14.7 years; 20 male). Among these patients, 20/25 (80.0%) were diagnosed with clear cell RCC (ccRCC), 4/25 (16.0%) with papillary RCC (pRCC) and 1/25 (4.0%) with undifferentiated RCC (uRCC); 11/25 (44.0%) underwent TKI therapy (*n* = 4 sunitinib, *n* = 4 cabozantinib, *n* = 1 lenvatinib/everolimus, *n* = 1 tivozanib, *n* = 1 axitinib); 12/25 (48%) underwent CI therapy (*n* = 9 ipilimumab/nivolumab, *n* = 1 nivolumab, *n* = 2 pembrolizumab); and 2/25 (8%) underwent both TKI and CI therapy (*n* = 2 cabozantinib/nivolumab) using standard dosages and no dose reduction during follow-up (Table 1).

### 3.2. Image Analysis

Most (24/25) of the patients underwent a follow-up [^18^F]PSMA-1007 PET/CT after initiation of therapy (mean 9.5 weeks). The remaining (1/25) patient was lost to follow-up and therefore excluded from the analysis. At PET_1_, 113 PSMA-positive metastases were analyzed in *n* = 18 patients (mean of 6.3 metastases/patient): *n* = 28 lymph node metastases, *n* = 28 osseous metastases, *n* = 8 hepatic metastases, *n* = 29 pulmonary metastases, *n* = 15 soft tissue metastases and *n* = 5 other metastases (*n* = 2 pancreas, *n* = 2 adrenal gland, *n* = 1 peritoneal). At PET_2_, 48 PSMA positive metastases were analyzed in *n* = 13 patients (mean of 3.7 metastases/patient): *n* = 13 lymph node metastases, *n* = 13 osseous metastases, *n* = 1 hepatic metastasis, *n* = 17 pulmonary metastases, *n* = 1 soft tissue metastasis, *n* = 3 other metastases (*n* = 1 adrenal gland, *n* = 2 peritoneal).

Overall, lymph node metastases showed a stable PSMA-radioligand uptake at PET_1_ and PET_2_ (PET_1_: median SUV_max_: 7.8 (Q1; 5.9; Q3: 10.4); PET_2_: median SUV_max_ 7.7 (Q1; 5.6; Q3: 16.0); *p* = 0.77 and PET_1_: median SUV_mean:_ 5.0 (Q1: 3.6; Q3: 6.8); PET_2_: median SUV_mean_ of 4.9 (Q1: 3.5; Q3: 10.2); *p* = 0.84). Bone metastases showed a significant increase of the median SUV_max_ (PET_1_: 6.4 (Q1: 4.5; Q3: 9.5); PET_2_: 12.4 (Q1: 7.0; Q3: 19.3); *p* = 0.03), whereas the median SUV_mean_ did not increase significantly (PET_1_: 5.0 (Q1: 3.5; Q3: 6.7); PET_2_: 8.6 (Q1: 4.9; Q3: 14.0); *p* = 0.09). In contrast, pulmonary metastases showed a significant increase of PSMA uptake in both median SUV_max_ (PET_1_: 4.5 (Q1: 3.5; Q3: 6.9); PET_2_: 8.1 (Q1: 5.8; Q3: 12.1); *p* = 0.004) and median SUV_mean_ (PET_1_: 3.1 (Q1: 2.2; Q3: 4.8); PET_2_: 5.2 (Q1: 4.0; Q3: 7.8); *p* = 0.003) (Table 2).

When analyzing uptake characteristics by the histological subtype, there was no significant change in the PSMA uptake seen for lymph node metastases of ccRCC (median SUV_max_ 7.9 vs. 12.5; *p* = 0.48; median SUV_mean_ 6.1 vs. 7.6; *p* = 0.66) or pRCC (median SUV_max_ 6.1 vs. 6.0; *p* > 0.99; median SUV_mean_ 3.7 vs. 3.9; *p* > 0.99). Bone metastases showed a significant increase of PSMA-radioligand uptake in ccRCC (median SUV_max_ 6.6 vs. 15.9; *p* = 0.008; median SUV_mean_ 5.0 vs. 11.3; *p* = 0.006) but not in pRCC (median SUV_max_ 6.2 vs. 6.0; *p* = 0.86; median SUV_mean_ 12.9 vs. 3.9; *p* = 0.38). Pulmonary metastases were only seen in patients with ccRCC. These metastases showed a significant increase of PSMA uptake (median SUV_max_ 4.8 vs. 8.1; *p* = 0.02; median SUV_mean_ 3.2 vs. 5.2; *p* = 0.004) (Table 3). There was only one patient with uRCC, who presented with a single lung metastasis with a distinct PSMA uptake (median SUV_max_ 2.8 vs. 2.3; median SUV_mean_ 1.7 vs. 1.3), but no analysis was conducted.

**Figure 1:**
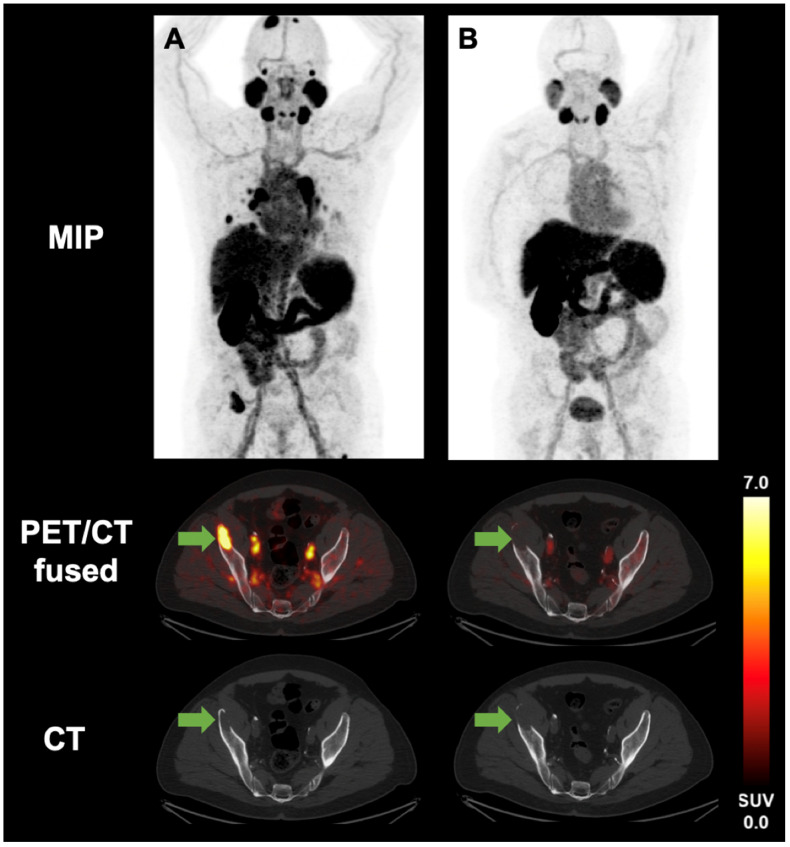
[^18^F]PSMA-1007 PET/CT of a 64-year-old male patient with metastatic ccRCC before (**A**) and after (**B**) therapy initiation. The arrow points to an osseous metastasis of the right iliac crest. Prior to systemic therapy (**A**) the SUV_max_ of the osseous metastasis was 11.0; after therapy initiation (**B**) no PSMA uptake was observed.

Figure 1 shows the imaging of an osseous metastasis of the right iliac crest of a 64-year-old male patient with metastatic ccRCC. Prior to systemic therapy, the SUV_max_ of the osseous metastasis was 11.0; after therapy initiation, no PSMA uptake was observed.

**Figure 2:**
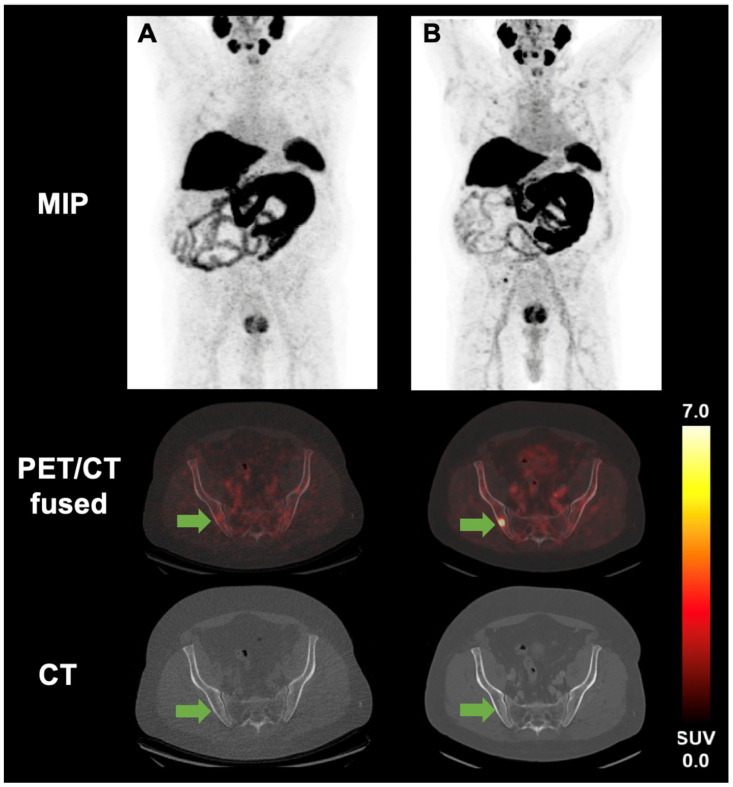
[^18^F]PSMA-1007 PET/CT of a 73-year-old male patient with metastatic pRCC before (**A**) and after (**B**) therapy initiation. The arrow points to an osseous metastasis of the right pelvis. Before treatment, the PSMA uptake was below the threshold of 4 (3.87) (**A**). After initiation of systemic therapy, on the follow-up scan, the patient presented with a newly diagnosed metastasis of the right pelvis and an increase in PSMA uptake of 13.6 (**B**). No CT–morphologic correlation was seen.

Figure 2 demonstrates the case of a 73-year-old male patient with metastatic pRCC and a newly diagnosed osseous metastasis of the right pelvis (SUV_max_ 13.6) without any CT–morphologic correlation.

## 4. Discussion

PSMA-radioligand uptake is increased in osseous and pulmonary metastases in RCC patients. Considering changes of PSMA uptake during systemic therapy, we observed an increased PSMA uptake in pulmonary (SUV_max_ and SUV_mean_) and osseous metastases (only SUV_max_), whereas lymph nodes showed no significant change. In our cohort, the number of PSMA positive metastatic lesions decreased from n = 116 to n = 50 during systemic therapy. A similar phenomenon was observed in patients with prostate cancer [21]. At this juncture, it is not yet possible to attribute such fluctuations in uptake to specific mechanisms, such as treatment resistance, progression, or immune escape, with any degree of certainty. The observed variations in tracer uptake may be indicative of a multitude of biological processes, including dynamic changes in neovasculature or the tumor microenvironment. There are no data available on how systemic therapy modifies PSMA expression levels and PSMA-radioligand uptake characteristics in RCC. However, it is known, for example, that TKIs change the FDG uptake due to interactions with GLUT1, which leads to false negative results [22]. Patients with prostate cancer present with increased PSMA expression, even though they respond to therapy biochemically and with reductions in lesion size [21]. Consequently, PSMA may not serve as a reliable marker for the aggressiveness of tumor lesions. Similar mechanisms should be considered when changes in PSMA expression in patients with mRCC are determined by using PSMA PET/CT. Consequently, such variations must be interpreted with caution. In the context of mRCC lesions, the increased PSMA uptake observed during systemic therapy may not necessarily be indicative of the prognosis or outcome. Conversely, it is conceivable that lesions exhibiting augmented PSMA uptake during systemic therapy could be those that evade systemic therapy. Further studies are needed to understand this observation and to ascertain whether these uptake patterns correlate with clinical outcomes, such as therapeutic efficacy or tumor adaption.

A subgroup analysis showed a significant increase in osseous metastases only in patients with ccRCC but not with pRCC. Although this study is limited by a low number of lesions in the pRCC subgroup, which may have contributed to this finding, the distinctly increasing bone uptake in ccRCC may also be explained by inherently different levels of PSMA expression within this histological subtype [8]. As previously reported, the strongest PSMA expression, as determined by immunohistochemistry, was seen in ccRCC, whereas pRCC showed a lower PSMA expression, in line with our PET findings for nodal and osseous metastases [8].

Since *non*-clear cell carcinoma showed PSMA uptake only in a small proportion of metastatic lesions, PSMA PET was only recommended for ccRCC by Yin et al. [23]. In contrast, in our cohort, pRCC also showed a strong uptake (SUV_max_ > 6.0); thus, the PSMA-positivity might be also explained by lesion size or pre-treatments. However, as only a few patients with pRCC were included, uptake values may be lower in a larger cohort. This limits the generalizability of our findings. Some studies reported that a positive PSMA PET/CT result was found to be strongly correlated with the intensity of PSMA expression on immunohistochemistry, particularly in the clear cell and chromophobe subtypes [24,25].

Nevertheless, the treatment regimens employed for different RCC subtypes are comparable. In contrast, *non*-ccRCC is associated with reduced remission rates and shorter progression-free survival [26]. Therefore, a more detailed understanding of PSMA dynamics in both ccRCC and *non*-ccRCC is necessary.

Additional basic science studies investigating the molecular and cellular mechanisms driving PSMA expression and modulation under systemic therapy in RCC are essential to complement clinical imaging data and improve biological understanding. The present analysis aims to describe these uptake patterns as a basis for such future research.

In patients with ccRCC, PSMA PET showed distinct advantages over [^18^F]FDG PET [23,27,28]. The PSMA PET-derived SUV_max_ was more effective in identifying tumor necrosis (AUC 0.85; *p* < 0.001) and adverse pathology (AUC 0.90; *p* < 0.001) than FDG PET/CT [27]. Aggarwal et al. demonstrated that compared to CT and [^18^F]FDG PET/CT, PSMA PET/CT performed better in detecting equivocal bone lesions in ccRCC, which is in line with our observations of a high PSMA expression (mean SUV_max_ > 6.0) in osseous lesions of ccRCC [15]. However, for the detection of hepatic lesions, CT showed better results, whereas for the detection of nodal metastases, detection rates were comparable between CT and PET [15]. Siva et al., however, reported a more rapid metabolic response on FDG PET in RCC patients under systemic therapy [11].

Studies using other tracers, such as the anti-CAIX antibody and radioligand [^89^Zr]Zr-girentuximab or [^111^In]In-girentuximab, have shown promising results in the imaging of ccRCC. They reported that treatment with a TKI (sorafenib) decreased the uptake of [^111^In]In-girentuximab [29,30,31].

Due to the low number of lesions, it was not possible to conduct statistical analyses for the other subgroups or metastatic regions. Therefore, changes of PSMA uptake in hepatic lesions or lesions in other visceral organs, e.g., adrenal glands or pancreas, were not evaluable. However, changes in PSMA uptake during systemic therapy might also be organ-specific and additional studies are warranted.

A baseline PSMA PET might be also helpful to stratify patients for specific therapies, e.g., a recent study demonstrated that PSMA PET can detect a polybromo-1 mutation (PBRM1), which has an impact on neoangiogenesis. These patients would benefit from PD-1 targeting immunotherapies [32]. Additionally, changes of angiogenesis could be also associated with differences in treatment response, especially to anti-angiogenic drugs such as TKIs [33,34]. Furthermore, a high PSMA uptake might allow patients to undergo PSMA targeted radiopharmaceutical therapy analogous to current clinical practice in prostate cancer patients [10]. This suggests the potential for a promising theranostic approach for RCC, particularly for cases where treatment options are limited. However, it is crucial to emphasize that the mechanism of action may differ substantially: in RCC, PSMA is mainly expressed in the tumor neovasculature rather than directly on the surface of tumor cells. This has the potential to influence therapeutic efficacy, biodistribution and radiation dose delivery. While this concept is biologically plausible, there is a paucity of clinical data at the moment. To date, only a single report has described PSMA radioligand therapy in RCC [35].

There are some limitations to our study. First, the cohort included is relatively small, which resulted in a low number of evaluable metastases. Metastases were not histologically proven. The retrospective design of the study constitutes a limitation, given that it results in a heterogeneous cohort with varying numbers of patients with histological subtypes included. However, the latter also enabled us to perform the first comparison of PSMA radioligand uptake between histological subtypes. The limited number of patients included in the study resulted in a corresponding limitation in the number of lesions that could be analyzed. Additionally, the overall analysis without consideration of histological subtype may have limited significance due to the heterogeneous nature of RCC subtypes. In particular, uRCC has the potential to introduce variability, thereby impacting the generalizability of the findings. Further analysis of individual lesions, coupled with additional histological investigations, is required. Furthermore, no lesion-based analysis was performed. Due to the challenges inherent in identifying and matching individual lesions across time points, especially in cases of small lesions, uptake quantification was conducted at the organ system level. Consequently, fluctuations in tracer uptake are indicative of mean values across affected organ compartments, rather than the dynamics of specific lesions. The observed changes in PSMA uptake reflect average uptake patterns within organ systems (e.g., lung) and do not permit conclusions about individual lesion biology. Whilst an increased tracer uptake may be suggestive of ongoing tumor activity, the absence of size progression, in conjunction with a paucity of lesion-specific follow-up data, precludes a definitive interpretation with regard to resistance or progression. This does limit the generalizability of the analysis but provides an important foundation for future studies.

It is important to keep in mind that PSMA uptake might also be nonspecific or occur in the context of inflammation [36]. A histopathological confirmation of PSMA expression or malignancy for individual lesions was not available in this retrospective analysis. The metastatic nature of the lesions was determined by means of multimodal imaging assessment, integrating findings from the PSMA PET and contrast-enhanced CT components, and was validated by follow-up imaging when available. Additionally, only lesions with clear metastatic morphology and PSMA-avid signals were included in the analysis.

Moreover, patients were not divided according to the systemic therapy they received. The mechanisms of action of CKI/TI differ substantially, and therefore it is predicted that they will exert divergent effects on PSMA expression and tracer uptake. Whilst this stratification would be highly relevant from biological and clinical viewpoints, the sample sizes within each treatment subgroup were too small to permit meaningful statistical comparisons when considering the histological subtype as well. Nevertheless, the influence of the administered therapeutic agents remains an important factor and is acknowledged as a limitation of the current study.

It is important to emphasize that the present study was not intended to assess treatment response; rather, it was specifically designed to characterize PSMA uptake patterns in metastatic lesions of RCC patients under systemic therapy. Consequently, no correlation with clinical outcome parameters such as RECIST criteria, progression-free survival, or overall survival was performed. Studies investigating the relevance of PSMA changes during systemic therapy on prognosis and outcome are underway. Nevertheless, the present analysis, being descriptive in nature, constitutes a significant initial step in the identification of organ- and histology-specific uptake behavior in the context of systemic therapy. Further research in this area is necessary to contribute to a more nuanced understanding of PSMA PET imaging in mRCC.

## 5. Conclusions

Changes of PSMA uptake were seen specifically in osseous and pulmonary metastases, but not in lymph node metastases, after initiation of systemic therapy in patients with mRCC. Here, the histological subtype of RCC was associated with distinct changes of PSMA-radioligand uptake. Although the underlying pathophysiological bases are not yet well understood, our data support the rationale to further evaluate the potential of PSMA as a biomarker in the context of systemic mRCC treatments, including immunotherapy.

## Figures and Tables

**Table 1:** Patient characteristics.

No.	Age	Sex	Histology	Systemic Therapy
1	47	F	ccRCC	Cabozantinib
2	76	F	ccRCC	Ipilimumab/Nivolumab
3	74	M	ccRCC	Everolimus/Lenvatinib
4	70	M	pRCC	Sunitinib
5	52	F	ccRCC	Cabozantinib
6	70	M	ccRCC	Sunitinib
7	44	M	ccRCC	Axitinib
8	24	M	pRCC	Nivolumab
9	42	M	uRCC	Ipilimumab/Nivolumab
10	73	M	ccRCC	Pembrolizumab
11	78	M	ccRCC	Sunitinib
12	64	M	ccRCC	Tivozanib
13	71	M	ccRCC	Pembrolizumab
14	73	M	pRCC	Sunitinib
15	85	M	ccRCC	Cabozantinib
16	73	F	ccRCC	Ipilimumab/Nivolumab
17	79	F	pRCC	Ipilimumab/Nivolumab
18	74	M	ccRCC	Ipilimumab/Nivolumab
19	71	M	ccRCC	Cabozantinib/Nivolumab
20	60	M	ccRCC	Cabozantinib
21	55	M	ccRCC	Ipilimumab/Nivolumab
22	57	M	ccRCC	Ipilimumab/Nivolumab
23	65	M	ccRCC	Ipilimumab/Nivolumab
24	65	M	ccRCC	Ipilimumab/Nivolumab
25 *	87	M	ccRCC	Cabozantinib/Nivolumab
Mean	65.2			
SD	14.7			

ccRCC, clear cell renal cell carcinoma; F, female; M, male; pRCC, papillary renal cell carcinoma; uRCC, undifferentiated renal cell carcinoma; * Patient 25 did not receive a follow-up PET/CT.

**Table 2:** PSMA uptake characteristics.

	PET_1_					PET_2_				
SUV_max_	Lymph Node	Bone	Liver	Lung	Soft Tissue	Lymph Node	Bone	Liver *	Lung	Soft Tissue
Median	7.8	6.4	26.0	4.5	7.0	7.7	12.4	31.7	7.5	2.0
Q1	5.9	4.5	17.8	3.5	5.6	5.6	7.0	31.7	5.3	2.0
Q3	10.4	9.5	36.9	6.9	12.2	16.0	19.3	31.7	11.8	2.0
SUV_mean_										
Median	5.0	5.0	14.3	3.1	5.2	4.9	8.6	18.2	5.2	1.3
Q1	3.6	3.5	10.7	2.2	3.3	3.5	4.9	18.2	4.0	1.3
Q3	6.8	6.7	27.2	4.8	8.7	10.2	14.0	18.2	7.8	1.3

Q1, 1st quartile; Q3, 3rd quartile; * liver with n = 1 metastasis.

**Table 3:** PSMA uptake characteristics by the respective histological subtype.

ccRCC	PET_1_					PET_2_				
SUV_max_	Lymph Node	Bone	Liver	Lung	Soft Tissue	Lymph Node	Bone	Liver	Lung	Soft Tissue
Median	7.9	6.6	21.2	4.8	8.3	12.5	15.9	n.a.	8.1	2.0
Q1	6.0	4.5	16.9	3.7	5.3	6.1	9.6	n.a.	5.3	n.a.
Q3	11.6	9.7	40.7	7.1	12.2	16.6	27.7	n.a.	12.1	n.a.
SUV_mean_										
Median	6.1	5.0	14.3	3.2	4.8	7.6	11.3	n.a.	5.2	1.3
Q1	3.8	3.2	10.7	2.4	3.2	3.8	7.3	n.a.	3.9	n.a.
Q3	8.1	6.6	27.2	4.9	8.7	11.4	17.3	n.a.	7.8	n.a.
**pRCC**	**PET_1_**					**PET_2_**				
**SUV_max_**	**Lymph Node**	**Bone**	**Liver**	**Lung**	**Soft Tissue**	**Lymph Node**	**Bone**	**Liver**	**Lung**	**Soft Tissue**
Median	n.a.	6.2	32.4	n.a.	5.2	6.0	n.a.	6.0	n.a.	n.a.
Q1	n.a.	5.2	n.a.	n.a.	n.a.	5.9	n.a.	n.a.	n.a.	n.a.
Q3	n.a.	7.2	n.a.	n.a.	n.a.	12.7	n.a.	n.a.	n.a.	n.a.
SUV_mean_										
Median	2.7	12.9	n.a.	n.a.	n.a.	3.4	3.9		4.1	n.a.
Q1	n.a.	9.3	n.a.	n.a.	n.a.	n.a.	3.7		n.a.	n.a.
Q3	n.a.	16.4	n.a.	n.a.	n.a.	n.a.	8.9		n.a.	n.a.
**uRCC**	**PET_1_**					**PET_2_**				
**SUV_max_**	**Lymph Node**	**Bone**	**Liver**	**Lung**	**Soft Tissue**	**Lymph Node**	**Bone**	**Liver**	**Lung**	**Soft Tissue**
Median	6.1	5.1	n.a.	2.8	7.0	6.0	n.a.	n.a.	2.3	n.a.
Q1	5.3	n.a.	n.a.	n.a.	n.a.	5.8	n.a.	n.a.	n.a.	n.a.
Q3	9.7	n.a.	n.a.	n.a.	n.a.	6.2	n.a.	n.a.	n.a.	n.a.
SUV_mean_										
Median	3.7	3.6	n.a.	1.7	5.3	3.9	n.a.	n.a.	1.3	n.a.
Q1	3.6	n.a.	n.a.	n.a.	n.a.	3.8	n.a.	n.a.	n.a.	n.a.
Q3	5.0	n.a.	n.a.	n.a.	n.a.	4.1	n.a.	n.a.	n.a.	n.a.

ccRCC, clear cell renal cell carcinoma; n.a., not applicable; pRCC, papillary renal cell carcinoma; Q1, 1st quartile; Q3, 3rd quartile; uRCC, undifferentiated renal cell carcinoma.

## Data Availability

The datasets used and/or analyzed during the current study are available from the corresponding author on reasonable request.

## Abbreviations

The following abbreviations are used in this manuscript:

ccRCC	clear cell renal cell carcinoma
CI	checkpoint inhibitor/inhibition
CT	computed tomography
F	female
M	male
mRCC	metastatic renal cell carcinoma
MRI	magnetic resonance imaging
PET	positron emission tomography
pRCC	papillary renal cell carcinoma
PSMA	prostate-specific membrane antigen
TKI	tyrosine kinase inhibitor/inhibition
uRCC	undifferentiated renal cell carcinoma
